# Anatomical Location of the Greater Palatine Foramen in the West Bengal Population: A Cross-Sectional Study of Adult Dry Skulls

**DOI:** 10.7759/cureus.108294

**Published:** 2026-05-05

**Authors:** Satabdi Sarkar, Samanwita Basak, Shahin S Khatun, Arpita Sarkar, Pallab K Saha

**Affiliations:** 1 Anatomy, All India Institute of Medical Sciences, Kalyani, Kalyani, IND; 2 Anatomy, Prafulla Chandra Sen (PCS) Government Medical College, Arambagh, IND; 3 Anatomy, Mahatma Gandhi Mission (MGM) Institute of Health Sciences, Mumbai, IND; 4 Anatomy, PKG Medical College and Hospital, Kolkata, India, Kolkata, IND; 5 Anatomy, Jalpaiguri Government Medical College and Hospital, Jalpaiguri, IND

**Keywords:** anatomical location, incisive fossa, mid sagittal plane of hard palate, posterior margin of hard palate, s: greater palatine foramen

## Abstract

Background: The greater palatine foramen (GPF) transmits the greater palatine vessels and nerve. Knowledge of its anatomical position is vital for maxillary anaesthesia in orofacial surgery, as well as for controlling bleeding during endoscopic sinus surgery, septorhinoplasty, posterior epistaxis, and palatal surgery.

Aim: This study was conducted to analyse the location of the GPF relative to various anatomical landmarks of the bony hard palate among adult dry skulls from West Bengal.

Methodology: A total of 104 dry skulls were examined. The vertical distance from the centre of the GPF to the posterior margin of the hard palate (PMHP), the oblique distance between the incisive fossa (IF) and the GPF, and the transverse distance between the mid-sagittal plane of the hard palate (MSHP) and the GPF were measured using digital vernier callipers. The location of the GPF in relation to the maxillary molar teeth and its direction of opening were noted in 41 skulls. Distances were compared between skulls with and without maxillary molar teeth. All data were tabulated and statistically analysed. The mean and standard deviation of the variables were calculated, and comparisons between sides and between skulls with and without maxillary molar teeth were performed using Student’s t-test (two-tailed, 95% confidence interval (CI)).

Results: The centre of the GPF was located 3.47 ± 0.94 mm and 3.55 ± 0.98 mm anterior to the PMHP on the right and left sides, respectively. The mean distance of the GPF from the IF was 37.27 ± 2.62 mm on the right side and 37.42 ± 2.73 mm on the left side, while the mean distance from the MSHP was 15.01 ± 1.81 mm on the right side and 14.88 ± 1.71 mm on the left side. The most common location of the GPF was medial to the third molar tooth, and the most common direction of opening was anteromedial. A significant variation in the distance from the MSHP to the GPF was observed between skulls with and without maxillary molar teeth.

Conclusions: These topographic data on the GPF aid surgeons in better evaluating the greater palatine nerve and vessels during oromaxillofacial surgery, particularly in the absence of maxillary molar teeth.

## Introduction

Anatomical precision on the location of the greater palatine foramen (GPF) is paramount in the fields of dentistry, maxillofacial surgery, and otorhinolaryngology [1-2]. The foramen is usually located posterolaterally on either side of the bony palate, which is formed by the palatine process of the maxilla and the horizontal plate of the palatine bone. It represents the lower end of the greater palatine canal, which transmits the greater palatine vessels and nerve from the pterygopalatine fossa [3]. The greater palatine nerve is often anaesthetised during various orofacial procedures. These regional blocks are used to improve recovery following maxillofacial surgery, particularly when general anaesthesia is contraindicated [4]. The greater palatine vessels may require ligation or cauterisation during endoscopic sinus surgery, septorhinoplasty, and in the management of posterior epistaxis [5-6]. The available data on the morphology and morphometry of the GPF in the literature from eastern India are limited to small sample sizes and single-centre studies. This study aims to elucidate the orientation and position of the greater palatine foramen in relation to surgically relevant anatomical landmarks and maxillary molars in adult dry skulls from West Bengal.

The primary objectives of this study were to determine the side-wise location of the GPF in relation to the maxillary molar teeth, incisive fossa (IF), posterior margin of the hard palate (PMHP), and mid-sagittal plane of the hard palate (MSHP). The secondary objective was to compare the anatomical location of the GPF between skulls with and without maxillary molar teeth.

## Materials and methods

This cross-sectional study was conducted in the Departments of Anatomy of three medical colleges in West Bengal from 2024 to 2025. The study was approved by the Institutional Ethics Committee (IEC) of Jagannath International Medical Sciences (JIMS) (approval number: JIMSH/IEC-2024/03-5). The minimum sample size was calculated using the following formula:

\[ N = \frac{Z^2 pq}{d^2} \]

With *p* = 0.5, *q* (1 − *p*) = 0.5, *Z* = 1.96 (95% confidence interval (CI)), and a precision of ±10%, the estimated minimum sample size was 96 skulls. However, a total sample of 104 skulls was used, following the approach of Kumar et al. [7].

Initially, 109 adult dry skulls of unknown sex were collected from the anatomy museums and the personal collections of students and faculty in the Departments of Anatomy of three medical colleges across different geographical locations in West Bengal. Of these, 104 skulls were selected after thorough examination for complete dentition, arch symmetry, absence of visible deformity or fracture of the hard palate, and an intact alveolar arch. Skulls with any pathological changes or damage to the hard palate, particularly in the regions of the GPF, IF, PMHP, and MSHP, were excluded.

The GPF was identified on the posterolateral aspect of the hard palate with reference to the mid-sagittal plane and posterior border. It is larger and located more anteriorly than the lesser palatine foramen (LPF).

The skulls were categorised into two groups:

(1) With maxillary molar teeth: when the bony socket of the maxillary molar, with or without the tooth, was present and clearly identifiable in the alveolar arch. (2) Without maxillary molar teeth: when the skulls lacked clear visualisation of the bony socket of the molar teeth, or when the alveolar bone had resorbed following tooth loss.

Of the 104 examined skulls, 41 had identifiable maxillary molar teeth and were included in the analysis of the position of the GPF. At the same time, the remaining 63 were excluded due to the absence of clear visualisation of the molar tooth and/or its bony socket and alveolar arch. So, the skulls with intact maxillary molar teeth were 41, while those without intact maxillary molar teeth were 63.

**Figure 1:**
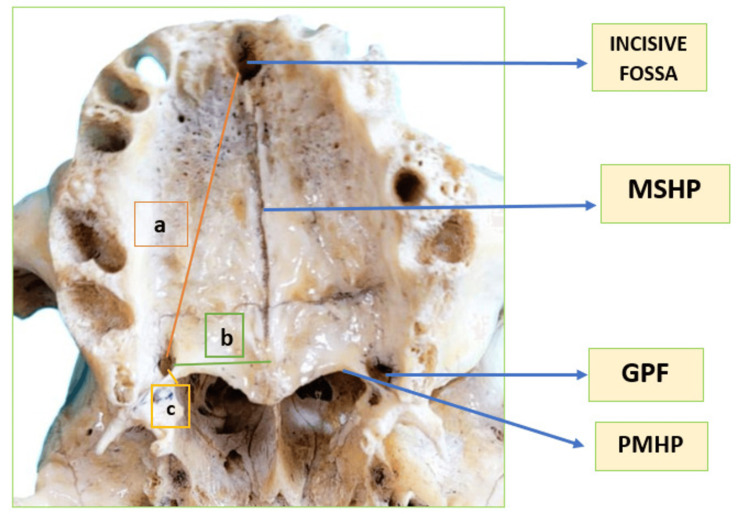
Measurement of the study parameters. (a) Distance from the GPF to the IF, (b) straight transverse distance from the centre of the GPF to the MSHP, and (c) distance from the centre of the GPF to the PMHP. Note: The figure is original and created by the author. MSHP, mid-sagittal plane of the hard palate; GPF, greater palatine foramen; PMHP, posterior margin of the hard palate

The following measurements were taken bilaterally (Figure 1): (a) distance from the GPF to the IF, (b) straight transverse distance from the centre of the GPF to the MSHP, and (c) distance from the centre of the GPF to the PMHP.

All measurements were taken using a sliding digital calliper with an accuracy of ±0.2 mm/0.01 mm, a measuring tape, and a thread. Measurements were obtained bilaterally and symmetrically. The measurements were recorded by two observers; where disagreements occurred, the mean of the two values was used. After all samples had been measured, 22 dry skulls from the three medical colleges were randomly selected, and the anatomical distances were measured by a third observer who had not participated in the initial data collection. Intraclass correlation coefficients (ICCs) were calculated using a two-way random-effects model for absolute agreement and single measurements (ICC (2,1)) [8]. The level of agreement between assessments was very high (ICC = 0.98-0.99).

The data were tabulated in an Excel spreadsheet and statistically analysed. Categorical variables were expressed as percentages. The mean, standard deviation, and range of continuous variables were calculated, and comparisons between the right and left sides and between skulls with and without maxillary molar teeth were performed using Student’s t-test (two-tailed, 95% confidence interval). The normality of data distribution within each group was assessed using the Shapiro-Wilk test. Homogeneity of variance between groups was evaluated using Levene’s test for equality of variances. A *P*-value of <0.05 was considered statistically significant. ICC was used to evaluate the level of agreement between measurements and repeated measurements of the same sample.

## Results

**Figure 2:**
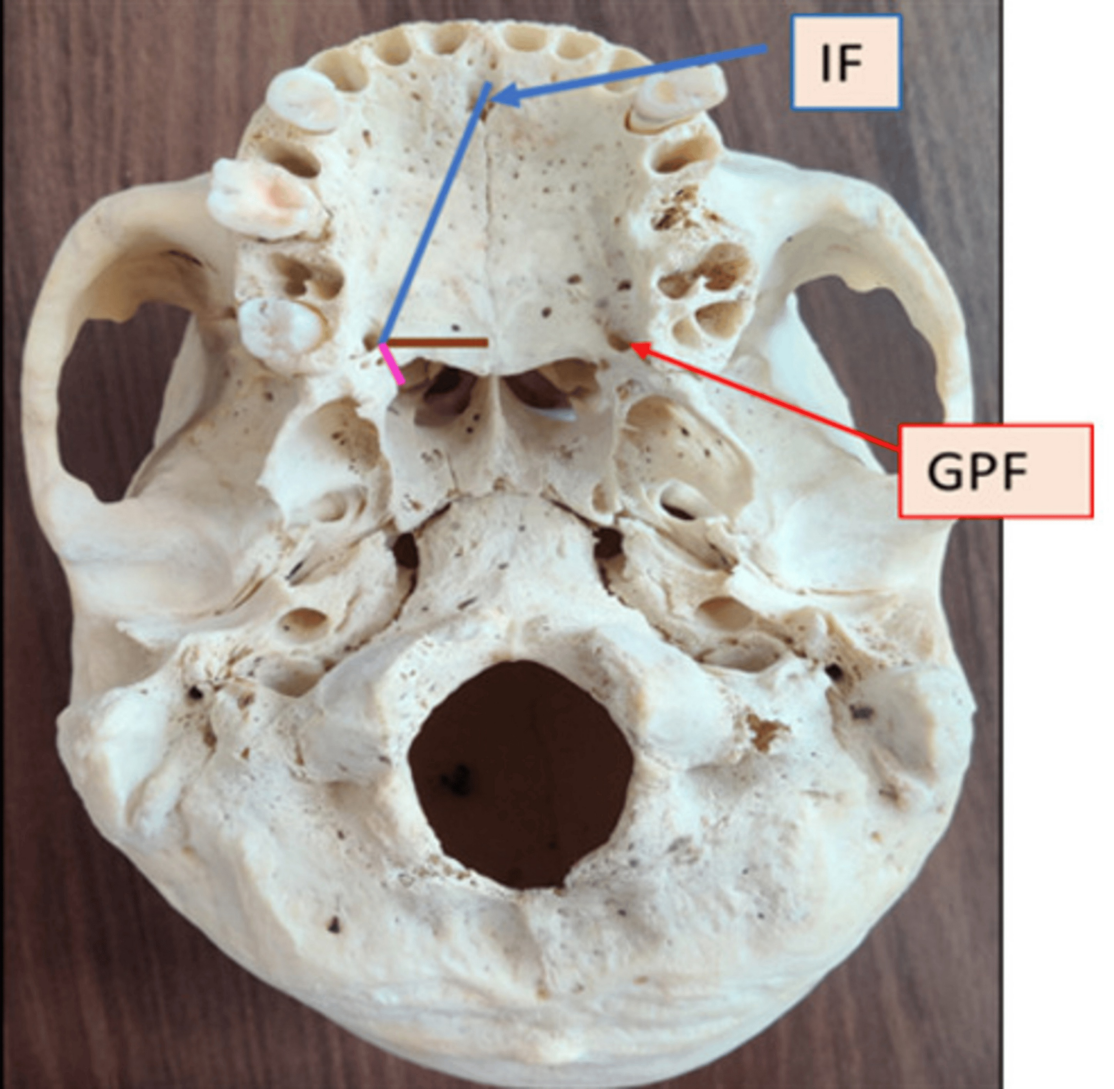
GPF located medial to the third maxillary molar. Note: The figure is original and created by the author. IF, incisive fossa; GPF, greater palatine foramen

**Figure 3:**
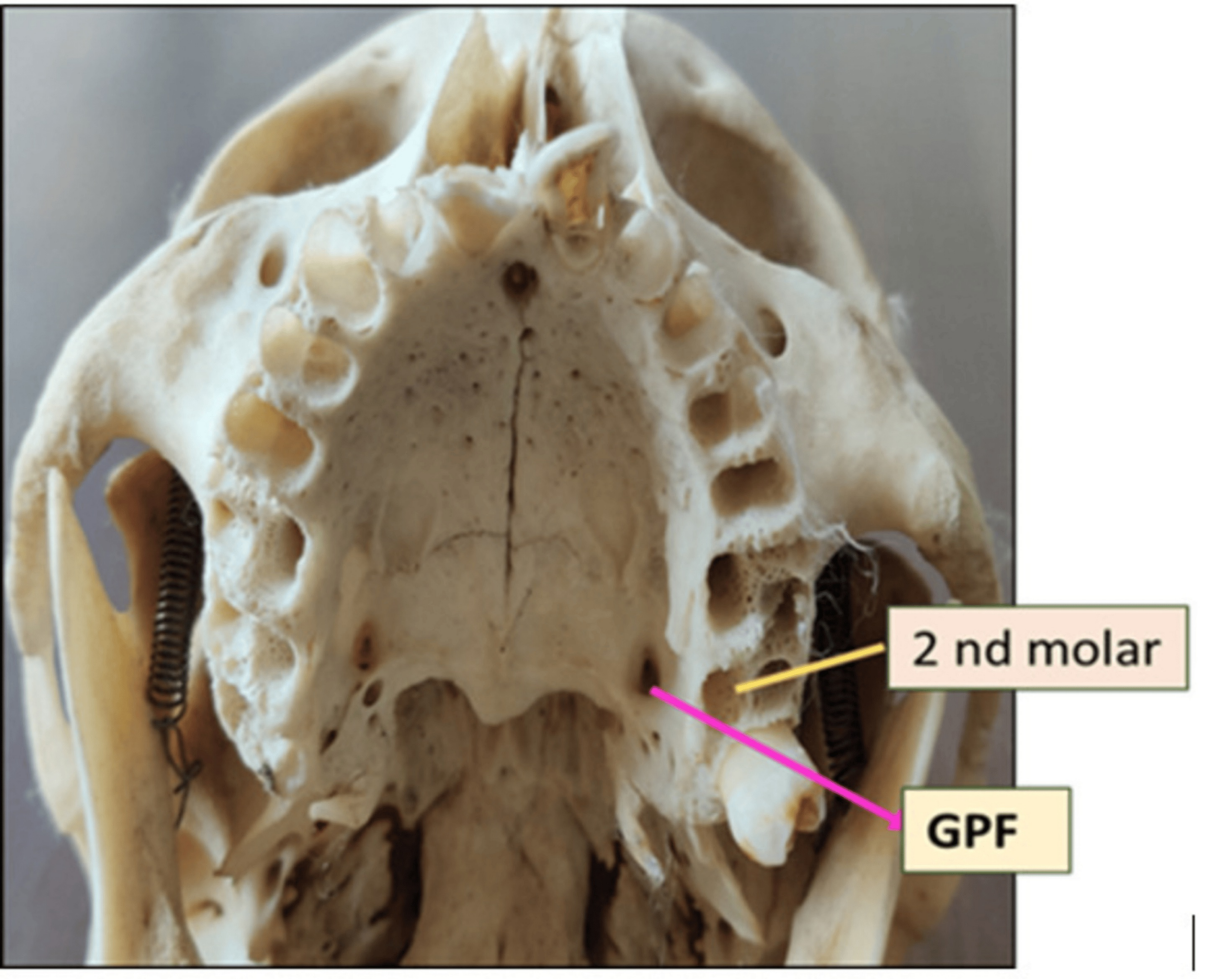
GPF located medial to the second maxillary molar. Note: The figure is original and created by the author. GPF, greater palatine foramen

After examining 104 adult skulls and applying the exclusion criteria, it was found that the most common location of the GPF was medial to the third molar, and the most common direction of opening was anteromedial in 41 skulls bilaterally (Table 1). In 36 skulls, the GPF was located medial to the third molar on both sides (Figure 2). In one skull, the GPF was located medial to the second molar on both sides (Figure 3).

**Table 1:** Position of the GPF in relation to the molar tooth or its bony socket. GPF, greater palatine foramen

Position of GPF in relation to the molar tooth or bony socket (*N* = 41)	Right side, *n* (%)	Left side, *n* (%)
Medial to the third molar tooth or bony socket	36 (87.80%)	36 (87.80%)
Between the second and third molar tooth or bony socket	4 (9.75%)	4 (9.75%)
Medial to the second molar tooth	1 (2.43%)	1 (2.43%)

Location of GPF

Table 1 shows that the GPF was located medial to the third molar tooth in 36 (87.80%) cases bilaterally. In four (9.75%) cases, the GPF was located between the second and third molar teeth, and in one (2.43%) case, it was located medial to the second molar. Bilateral symmetry in the position of the GPF in relation to the molar teeth was observed in this study.

Topographic relationships of the GPF 

In Table 2, all parameters show excellent reliability, with ICC values ranging from 0.980 to 0.989. The Shapiro-Wilk test confirmed normal distribution of the data (*P* > 0.05), while Levene’s test indicated homogeneity of variance (*P* > 0.05). These findings indicate that the data satisfy the assumptions for parametric statistical analysis and reflect a high level of measurement consistency. 

**Table 2:** Intraclass correlation coefficients (ICC), normality (Shapiro-Wilk test), and homogeneity of variance (Levene’s test) for all variables. Shapiro-Wilk test: *P* > 0.05 → normal distribution *P* < 0.05 → non-normal distribution Levene’s test: *P* > 0.05 → homogeneity met *P* < 0.05 → homogeneity not met MSHP, mid-sagittal plane of the hard palate; GPF, greater palatine foramen; PMHP, posterior margin of the hard palate; IF, incisive fossa

Parameters	ICC (95% CI)	Shapiro-Wilk (W)	*P*-value	Normality	Levene’s test (F)	*P*-value	Homogeneity
GPF to PMHP	0.980 (0.976-0.989)	0.99	0.56	Normal	0.01	0.97	Met
GPF to MSHP	0.989 (0.982-0.992)	0.98	0.34	Normal	0.91	0.34	Met
GPF to IF	0.989 (0.987-0.994)	0.99	0.36	Normal	0.46	0.49	Met

Table 3 shows that the largest distance from the GPF was to the IF, and the smallest was to the PMHP. No statistically significant difference was observed between the right and left sides for GPF to PMHP and GPF to IF (*P* > 0.05). However, a significant difference was observed in the GPF-to-MSHP distance between the right and left sides.

**Table 3:** Topographic parameters of GPF in relation to the surrounding anatomical bony landmarks of the hard palate (n = 104). MSHP, mid-sagittal plane of the hard palate; GPF, greater palatine foramen; PMHP, posterior margin of the hard palate; IF, incisive fossa; SD, standard deviation; CI, confidence interval

Parameters (*n* = 104)	Side	Mean (mm)	SD	Maximum (mm)	Minimum (mm)	Mean diff	*t*-value	*P*-value	95% CI
GPF to PMHP	Right	3.47	0.94	5.9	1.5	0.08	1.1	>0.05 (insignificant)	-0.24 to 0.08
	Left	3.55	0.98	7.3	1				
GPF to IF	Right	37.27	2.62	42.8	30.1	0.15	1.4	>0.05 (insignificant)	-0.40 to 0.10
	Left	37.42	2.73	43.8	30.9				
GPF to MSHP	Right	15.01	1.81	19.9	11	0.13	1.3	0.03 (significant)	0.05 to 0.31
	Left	14.88	1.71	19.1	11.5				

Morphometric comparison based on the presence or absence of maxillary molars

The study sample was divided into two groups based on the presence or absence of intact maxillary molar teeth: specimens with intact maxillary molar teeth (41, 39.42%) and specimens without intact maxillary molar teeth (63, 60.58%).

Tables 4-5 show a statistically significant difference in the GPF-to-MSHP distance between skulls with and without maxillary molar teeth on both sides. The distances to the other two anatomical landmarks, i.e., GPF to PMHP and GPF to IF, did not differ significantly between skulls with and without maxillary molar teeth on both sides.

**Table 4:** Morphometric parameters for comparison of dimensions between skulls with and without maxillary molar teeth (right side). MSHP, mid-sagittal plane of the hard palate; GPF, greater palatine foramen; PMHP, posterior margin of the hard palate; IF, incisive fossa; SD, standard deviation; CI, confidence interval

Parameters		Mean	SD	Mean diff	*t*-value	*P*-value	95% CI
GPF to MSHP	With the maxillary molar teeth	14.40	1.68	1.01	2.84	0.005 (Significant)	0.31 to 1.71
	Without the maxillary molar teeth	15.40	1.80				
GPF to PMHP	With the maxillary molar teeth	3.63	1.07	0.26	1.43	>0.05 (Insignificant)	-0.11 to 0.63
	Without the maxillary molar teeth	3.36	0.83				
GPF to IF	With the maxillary molar teeth	37.18	2.38	0.14	0.28	>0.05 (Insignificant)	-0.91 to 1.19
	Without the maxillary molar teeth	37.33	2.79				

**Table 5:** Morphometric parameters for comparison of dimensions between skulls with a maxillary molar tooth and those without a maxillary molar tooth (left side). MSHP, mid-sagittal plane of the hard palate; GPF, greater palatine foramen; PMHP, posterior margin of the hard palate; IF, incisive fossa; SD, standard deviation; CI, confidence interval

Parameters		Mean	SD	Mean diff	*t*-value	*P*-value	95% CI
GPF to MSHP	With the maxillary molar teeth	14.46	1.56	0.73	2.18	0.03 (Significant)	0.07 to 1.39
	Without the maxillary molar teeth	15.19	1.74				
GPF to PMHP	With the maxillary molar teeth	3.6	0.96	0.13	0.66	>0.05 (Insignificant)	-0.26 to 0.52
	Without the maxillary molar teeth	3.46	1.02				
GPF to IF	With the maxillary molar teeth	37.32	2.66	0.26	0.49	>0.05 (Insignificant)	-0.82 to 1.34
	Without the maxillary molar teeth	37.59	2.75				

## Discussion

The GPF is located at the posterolateral aspect of the hard palate. This foramen transmits the greater palatine nerve and vessels. The nerve is a branch of the pterygopalatine ganglion and carries both general sensory fibres from the maxillary nerve and parasympathetic fibres from the nerve of the pterygoid canal. It requires anaesthesia for procedures involving the oral cavity and maxillary (upper) teeth. The greater palatine artery is a branch of the descending palatine artery, itself a terminal branch of the maxillary artery. It passes through the greater palatine canal and anastomoses with the sphenopalatine artery, contributing to the blood supply of the hard palate and nasal septum [9-13].

Precise localisation of the GPF and the structures transmitting through it is essential for successful palatal anaesthesia and surgical procedures involving the posterior maxilla. Clinicians generally locate the GPF to access the greater palatine nerve and vessels quickly and effectively in relation to the third molars [9-10]. However, in the absence of third molars or in edentulous patients, identifying the GPF clinically is quite challenging for surgeons. The present study aimed to evaluate the anatomical location of the GPF in adult dry skulls and document its variations relative to commonly used anatomical landmarks.

Position of the greater palatine foramen in relation to predetermined anatomical landmarks

In our study, the GPF can be accurately located anterolaterally by identifying the IF, an easily identifiable landmark, laterally from the MSHP, which is felt as a midline vertical depression on the hard palate, and from the junction of the hard and soft palate at the PMHP. The use of multiple anatomical reference points simplifies the task of locating the GPF for anaesthetists and reduces complications [14]. The IF bears a foramen, the incisive foramen, which is a funnel-shaped opening in the bone of the hard palate. The incisive foramen is situated immediately behind the incisor teeth. In our study sample, the GPF was located approximately 37 mm posterolateral to the IF, 3.5 mm anterior to the PMHP, and 15 mm lateral to the MSHP. These distances are comparable with study results from different geographical regions [6-7,13-17]. Table 6 depicts these study results. 

**Table 6:** Comparison of this study’s results with previous studies from different geographical regions.

Study	Geographical location	IF to GPF (mm)		MSHP to GPF (mm)		PMHP to GPF	
		Right side	Left side	Right side	Left side	Right side	Left side
Present study	West Bengal, Eastern India	37.27 ± 2.62	37.42 ± 2.63	15.01 ± 1.81	14.88 ± 1.71	3.47 ± 0.94	3.55 ± 0.98
Kumar et al. [7]	India	40.68	36.32				
Saralaya and Nayak [13]	South India	37.2 ± 0.29	37.4 ± 0.3	14.7 ± 0.15	14.7 ± 0.14	4.2 ± 0.139	4.2 ± 0.133
Sushobhana et al. [14]	North India			13.38 ± 1.38	13.58 ± 1.21	3.38 ± 1.30	3.34 ± 1.25
Anjankar et al. [15]	Central India	36.2	35.7	15.4	15.1	3.5	3.3
Voljevica et al. [16]	Bosnia and Herzegovina	40.12 ± 2.19	40.34 ± 2.08	15.80 ± 1.28	15.86 ± 1.19	4.00 ± 1.07	4.35 ± 1.34
Ilayperuma et al. [6]	Sri Lanka			15.2 ± 1.24	15.28 ± 1.06	4.52 ± 1.86	4.56 ± 1.03
Chrcanovic and Custódio [17]	Brazil	36.21 ± 3.16	36.52 ± 3.34	14.68 ± 1.56	14.44 ± 1.43	3.39 ± 1.11	

Position of the greater palatine foramen in relation to the third molar tooth

In our study, we found that the most common location of GPF was medial to the third maxillary molar among skulls with the maxillary tooth, and the most common direction of opening was anteromedial. These findings are similar to all other studies [12-15], although a few Asian studies found the antero-medial direction of the GPF in half of the cases [14-16]. Few studies mentioned that the foramen was located between the second and third molars or distal to the third molar [13]. Such variability highlights the importance of considering individual anatomical differences during clinical procedures rather than relying solely on textbook descriptions.

The molar-based localisation of the GPF is particularly useful for dental practitioners during greater palatine nerve block, especially in dentate individuals. In edentulous patients, the need for additional anatomical reference points emerges.

Shape and morphology of the greater palatine foramen

The oval and round shapes of the foramen were the most common in our study. Similar observations have been reported by Chrcanovic and Custódio [17] as well as Ikuta et al. [18]. Variations in the shape and size of the foramen may affect needle insertion during anaesthesia. A narrow or slit-like foramen may pose technical difficulties during anaesthesia or surgical manipulation, whereas a wider foramen may increase the risk of vascular injury. Therefore, awareness of such morphological variations is essential to minimise complications [17].

Laterality and symmetry

Our study also showed no significant side-to-side variation in the location of the GPF relative to the predetermined anatomical landmarks among all study samples, as well as among the skulls with and without maxillary molar teeth in the case of two predetermined anatomical landmarks. This result will help the surgeon to locate the greater palatine nerve and vessels in case of a unilaterally distorted or pathologically affected hard palate.

The only significant variation has been observed in the distances from the MSHP between skulls with and without the maxillary molar tooth. These results will help the surgeon consider other anatomical landmarks, such as IF and PMHP, to determine the location of GPF, especially in the absence of a maxillary tooth or in edentulous patients.

These findings are comparable to previous studies that reported general bilateral symmetry with occasional asymmetry [14-15]. Clinically, these rare asymmetries may explain unilateral failure of palatal anaesthesia and support the need for cautious bilateral assessment during procedures [17].

Comparison of the study results with the CBCT study

Our study shows that the mean distance between the GPF and IF is 37.27 ± 2.62 mm on the right and 37.42 ± 2.73 mm on the left side. A similar result was found in the study by Viveka and Kumar [19], using CT scans in adult individuals from Kerala, India (GPF to IF on the right side was 39.76 ± 2.47 mm and on the left side was 37 ± 2.39 mm). They also observed the position of the GPF adjacent to the third molar tooth in 56% of cases. In contrast, in our study, we observed it in 87.8% of cases. Our study is in concurrence with another CBCT study conducted on fifty adult Brazilian patients. They observed that the average distance of the GPF from the midline maxillary suture (MMS) was 15.3 mm, which was 15.01 ± 1.81 mm on the right side and 14.88 ± 1.71 mm on the left side in our study [20].

Ethnic and population variations

Our study results demonstrate certain differences when compared with other ethnic populations. Variations in the position and morphometry of the GPF have been documented across African, Caucasian, and Asian populations [6,14-18]. These differences may be due to genetic factors, craniofacial morphology, and environmental influences affecting maxillary development. The present study thus contributes valuable population-specific data, which is essential for clinicians practising in the concerned region.

Clinical implications

Accurate localisation of the GPF is crucial for the success of greater palatine nerve block, palatal surgeries, cleft palate repair, periodontal procedures, orthognathic surgery, and placement of maxillary implants. Detailed anatomical knowledge reduces the risk of complications such as haemorrhage, nerve injury, and inadequate anaesthesia [9,15]. The results of the present study provide practical guidelines for clinicians to improve procedural accuracy and patient safety.

Limitations of the study

The study was conducted on dry adult skulls, which lack soft-tissue representation; hence, the study results need to be validated in living patients. The age and sex of the majority of the skulls were unknown, which may have influenced their palatal dimensions. Additionally, the convenience sampling method may not fully represent the general population, which is a limitation of the present study. The lack of high-resolution CT scan data among living individuals of specific age groups, and the regional origin of the skulls, may limit the generalizability of the findings.

Scope for future research

Future studies may include radiological evaluation using cone-beam computed tomography (CBCT) in living subjects from the eastern zone to correlate osseous findings with clinical landmarks. Studies involving sex determination, age-related changes, and a larger sample size would further enhance the clinical applicability of the data.

Strengths of the study

The use of multiple clinically relevant direct osteological examinations of anatomical landmarks on adult dry skulls from a specific geographic location strengthens our study. Bilateral measurements were performed using standardised anatomical landmarks, and each measurement was independently recorded. ICC was used to evaluate the level of agreement between the measurement and re-measurement of the same sample; thus, it further increases the reliability of the study results. The topographical comparisons between skulls with and without maxillary teeth further opened another direction for our study, helping the clinician locate the GPF correctly.

## Conclusions

The GPF can be located antero-laterally relative to the incisive fossa, the mid-sagittal plane, and the posterior margin of the hard palate. The mean distances are consistent with the other study results of this geographical area. Side-wise variations are not significant. As significant differences in the distance from the IF and PMHP to the GPF among the two varieties of the skull, with and without maxillary molar teeth, were not found, and only the MSHP distance showed a statistically significant difference, the data of the GPF distance from MSHP may serve as a reliable reference for locating the greater palatine nerve and vessels among the edentulous subjects or in individuals without a molar tooth.

## References

[1] Methathrathip D, Apinhasmit W, Chompoopong S, Lertsirithong A, Ariyawatkul T, Sangvichien S (2005). Anatomy of greater palatine foramen and canal and pterygopalatine fossa in Thais: considerations for maxillary nerve block. Surg Radiol Anat.

[2] Baddour HM, Hubbard AM, Tilson HB (1979). Maxillary nerve block used prior to awake nasal intubation. Anesth Prog.

[3] Douglas R, Wormald PJ (2006). Pterygopalatine fossa infiltration through the greater palatine foramen: where to bend the needle. Laryngoscope.

[4] Kulkarni MR, Shettar LG, Bakshi PV, Thakur SL (2018). A novel clinical protocol for the greater palatine compression suture: a case report. J Indian Soc Periodontol.

[5] Sharma NA, Garud RS (2013). Greater palatine foramen--key to successful hemimaxillary anaesthesia: a morphometric study and report of a rare aberration. Singapore Med J.

[6] Ilayperuma I, Nanayakkara G, Palahepitiya N (2014). Morphometric evaluation of the greater palatine foramen in adult Sri Lankan skulls. Int J Morphol.

[7] Kumar N, Bhaskar R, Shidaraddi A, Lewis M Glenda (2025). An analytical study of the greater palatine foramen in adult human skulls using anatomical planes for clinical considerations. Transl Res Anat.

[8] Koo TK, Li MY (2016). A guideline of selecting and reporting intraclass correlation coefficients for reliability research. J Chiropr Med.

[9] Kim DW, Tempski J, Surma J (2023). Anatomy of the greater palatine foramen and canal and their clinical significance in relation to the greater palatine artery: a systematic review and meta-analysis. Surg Radiol Anat.

[10] Singh D, Patnaik P, Gupta N (2019). A study of the anatomical variation and clinical considerations of greater palatine foramen in adult human skulls of north Indian population. Int J Health Sci Res.

[11] Hafeez NS, Ganapathy S, Sondekoppam R, Johnson M, Merrifield P, Galil KA (2015). Anatomical variations of the greater palatine nerve in the greater palatine canal. J Can Dent Assoc.

[12] Aoun G, Zaarour I, Sokhn S, Nasseh I (2015). Maxillary nerve block via the greater palatine canal: an old technique revisited. J Int Soc Prev Community Dent.

[13] Saralaya V, Nayak SR (2007). The relative position of the greater palatine foramen in dry Indian skulls. Singapore Med J.

[14] Sushobhana Sushobhana, Mishra SR, Singh S, Passey J, Singh R, Sinha P (2015). Anatomical study and clinical considerations of greater palatine foramen in adult human skulls of North Indian population. Int J Anat Radiol Surg.

[15] Anjankar P, Gupta DS, Nair S (2014). Analysis of position of greater palatine foramen in central Indian adult skulls: a consideration for maxillary nerve block. Indian J Pharm Biol Res.

[16] Voljevica A, Talović E, Kapur E (2024). Morphometric analysis of the greater palatine foramina in the Bosnia and Herzegovina population. Acta Med Acad.

[17] Chrcanovic BR, Custódio AL (2010). Anatomical variation in the position of the greater palatine foramen. J Oral Sci.

[18] Ikuta CR, Cardoso CL, Ferreira-Júnior O, Lauris JR, Souza PH, Rubira-Bullen IR (2013). Position of the greater palatine foramen: an anatomical study through cone beam computed tomography images. Surg Radiol Anat.

[19] Viveka S, Kumar M (2016). Radiological localization of greater palatine foramen using multiple anatomical landmarks. MOJ Anat Physiol.

[20] Tomaszewska IM, Tomaszewski KA, Kmiotek EK, Pena IZ, Urbanik A, Nowakowski M, Walocha JA (2014). Anatomical landmarks for the localization of the greater palatine foramen--a study of 1200 head CTs, 150 dry skulls, systematic review of literature and meta-analysis. J Anat.

